# Immediate Bilateral Sequential Keratoplasty in a 13‐Year‐Old Boy With Perforated Corneal Ulcer Secondary to Exposure Keratopathy

**DOI:** 10.1002/ccr3.71273

**Published:** 2025-10-15

**Authors:** Javad Sadeghi, Mohammadreza Sedaghat, Mitra Karimi Amir Abadi, Mehrdad Motamed Shariati

**Affiliations:** ^1^ Eye Research Center Mashhad University of Medical Sciences Mashhad Iran

**Keywords:** cornea, corneal transplantation, exposure keratopathy, intensive care units, penetrating keratoplasty

## Abstract

To present the challenging scenario of a 13‐year‐old patient in the ICU with bilateral corneal perforations secondary to EK, who underwent immediate sequential bilateral penetrating keratoplasty. Furthermore, we explore the treatment outcomes of this approach. A 13‐year‐old boy presented with decreased vision in both eyes. Upon ophthalmic examination, we identified bilateral perforated corneal ulcers with iris prolapse and a flat anterior chamber (AC). Due to the bilateral corneal perforations and the heightened risk of infectious endophthalmitis, immediate bilateral sequential penetrating keratoplasty (PKP) was carried out for the patient the day after presentation to our emergency room (ER). Over the 6‐month follow‐up period, no signs of graft rejection or infection were observed. This is the first reported case of immediate sequential bilateral PK. In emergent situations like bilateral corneal perforation, sequential bilateral surgery proves to be a reasonable and effective option.

**Summary**

- Severe exposure keratopathy can lead to rapid corneal perforation, necessitating urgent surgical intervention.
- Immediate bilateral sequential keratoplasty restores ocular integrity and highlights the importance of timely management in such high‐risk cases, particularly in children.

## 1 Introduction

Exposure keratopathy (EK) is a well‐known corneal disorder due to dysregulation of the ocular surface homeostasis secondary to eyelid closure abnormality and prolonged corneal exposure. The main etiologies include lagophthalmos, lid malposition, proptosis, neurologic, and neurotrophic disease [1]. EK was reported in 10%–60% of critically ill patients in the intensive care units (ICU) [1]. Decreased blink rate and incomplete eye closure are the main initiators. Regarding the severity and exposure time, a spectrum of signs has been reported, from inferior corneal superficial punctate epithelial erosions to severe stromal thinning, vascularization, and corneal perforation [2, 3]. As a common finding in intensive care unit patients (ICU), EK is reported in about half of the patients, and it can lead to severe vision loss or even predispose patients to infectious keratitis. Therefore, it is vital to consider EK a significant threat to ICU patients, and prophylactic considerations should be considered. There are several ways to reduce the incidence of EK, such as moisture chambers, lubricants, eyelid taping, and temporary tarsorrhaphy [4].

Vision loss is the most severe complication of EK. Lubricating agents, amniotic membrane transplantation, and scleral contact lenses are some of the primary options for managing the early stages of EK. However, corneal transplantation should be considered in more severe conditions such as corneal thinning, vascularization, haziness, and perforation [1, 2]. Corneal blindness is one of the most common causes of reversible blindness. It can be managed with penetrating keratoplasty (PKP), which involves a full‐thickness corneal replacement by a healthy donor.

In this report, we present a case of a 13‐year‐old patient who was hospitalized in the ICU with bilateral corneal perforation secondary to EK. The patient underwent immediate sequential bilateral PKP, and we also examined the resulting outcomes.

## 2 Case History and Examination

A 13‐year‐old boy presented with decreased vision in both eyes that had developed approximately 1 week before admission. According to the patient's family, he had been hospitalized in the intensive care unit (ICU) in Afghanistan for 1 week due to an episode of unconsciousness following an acute abdomen, for which he underwent an emergency laparotomy around 3 weeks earlier. Unfortunately, no medical records were available to confirm the details of his condition or treatment, and the history was based entirely on family reporting. The patient noticed visual loss after regaining consciousness. His visual acuity was hand motion detection in both eyes. Upon ophthalmic examination, we noticed bilateral perforated corneal ulcers with iris prolapse and a flat Anterior Chamber (AC). In both eyes, the perforation involved the cornea's central and inferior paracentral region, measuring approximately 4.5 mm in diameter. The margins were infiltrated. The lens in both eyes appeared clear and in place, with no evidence of cataract formation.

## 3 Methods

**FIGURE 1:**
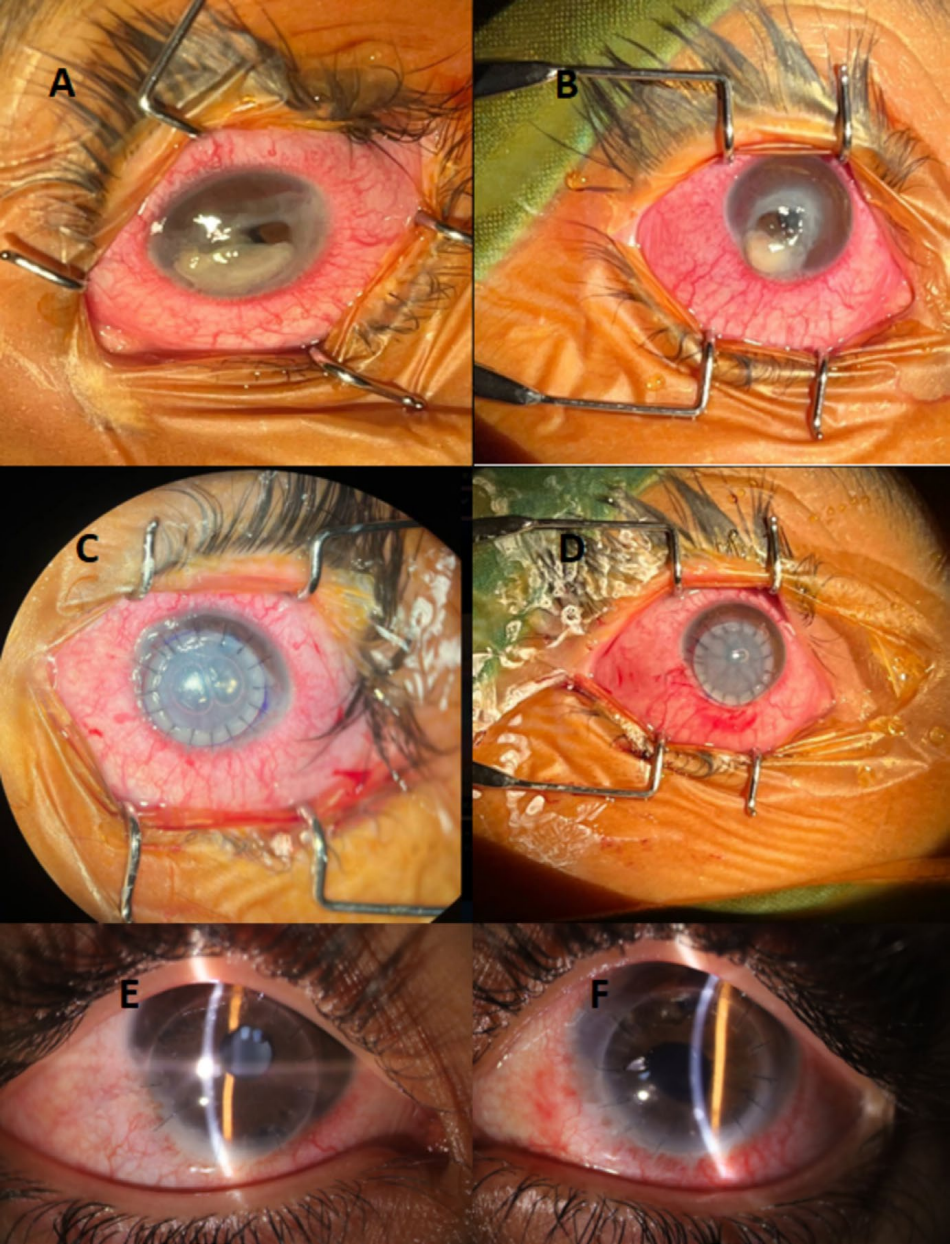
The slit photograph of the patient's eyes shows bilateral perforated corneal ulcer (top row, A and B) at the time of presentation, stable decentered graft at the end of PKP (middle row, C and D), and clear corneal grafts at the 6‐month follow‐up (E and F).

Regarding the bilateral perforated corneas and the increased risk of infectious endophthalmitis, immediate sequential bilateral PKP, with each eye operated on using a completely independent surgical setup, including distinct instruments, drapes, gloves, and solutions, under general anesthesia was performed for the patient the day after the presentation to our emergency room (ER) (Figure 1). The graft size was 8.5 mm, and the host cornea was trephined at 8.0 mm. The recipient bed was carefully cleaned, and the infiltrated margins were excised. Intraoperatively, the crystalline lens was intact and clear in both eyes. Therefore, no lens extraction or intraocular lens (IOL) implantation was performed. There were no significant complications during the procedures. Due to the decentered corneal perforation and a relatively healthy corneal rim, the graft was also decentered. Postoperatively, B‐scan ultrasonography revealed no evidence of posterior segment complications such as retinal detachment or choroidal effusion. The medications prescribed at discharge included oral prednisolone (15 mg daily), mycophenolate mofetil 500 mg every 12 h, topical moxifloxacin, and tropicamide. Topical corticosteroids were initially withheld due to concerns about active infection and were introduced cautiously once the infection was controlled. Systemic immunomodulation was continued and monitored throughout the follow‐up period to optimize graft survival and minimize complications.

## 4 Conclusion and Results

No signs of graft rejection or infection were observed during the follow‐up period. The intraocular pressure, measured with Goldman applanation tonometry, remained within normal limits for both eyes. At the last follow‐up visit, conducted 6 months after the initial presentation, clear grafts were noted, and the visual acuity was measured at 5/10, measured with the Snellen chart, for both eyes (Figure 1).

## 5 Discussion

The nurses and staff of the ICU devote close attention to vital signs, life‐threatening conditions, and stabilizing the patient's hemodynamics because of the poor state of the patients admitted. Patients might be disregarded for easily avoidable ophthalmic complications, such as EK. A further contributing aspect is a lack of knowledge regarding the risk factors and preventative strategies for EK. Chen et al. in a meta‐analysis in 2023, showed that the prevalence of EK in ICU patients was 34% and 40% in adults and children, respectively, which was compatible with the results of Rosenberg's study [5, 6]. Sedation, mechanical ventilation, lagophthalmos, chemosis, eye blinks less than five times per minute, lower Glasgow Coma Scale (GCS) score, and higher Acute Physiology and Chronic Health Evaluation (APACHE) II score are significant risk factors associated with EK [5].

Exposure keratopathy can manifest with varying degrees of severity. In cases of mild EK, individuals may experience photophobia, blurred vision, and discomfort due to corneal epithelial erosions. Conversely, severe, overlooked, and inadequately treated instances can lead to persistent corneal scarring, vision impairment, and significant complications like corneal perforation. Microbial keratitis poses the greatest threat as it can culminate in blindness, endophthalmitis, and corneal perforation [4, 5, 6]. Most EK cases are mild, and bilateral sterile perforation, as seen in the patient's case, is exceedingly rare.

While most patients have mild to moderate severity, managing severe cases of exposure keratopathy can be challenging. Prevention and primary conservative measures, such as artificial tears and lubricant ointments, are crucial in effective management. A study involving 50 consecutive ICU patients receiving propofol or neuromuscular blockade showed that using lubricating ointment every 4 h significantly reduced exposure keratopathy compared to passive eyelid closure alone [7]. Various methods, such as tarsorrhaphy, moisture chambers, and eyelid taping, can help reduce tear evaporation. A study with 207 mechanically ventilated and intubated children found no added benefit from using a moisture chamber over lubricating ointment every 6 h [8].

One of the non‐traumatic causes of corneal perforations that results in ocular morbidity and significant vision loss is severe exposure keratopathy. Corneal perforations must be treated immediately to preserve the cornea's anatomic integrity and avoid consequences like endophthalmitis or subsequent glaucoma. The perforation's size, location, and underlying reason will determine the optimal treatment approach. Treatment options for corneal perforation range from temporary methods like utilizing bandage contact lenses and gluing to permanent options like corneal transplantation [9]. Corneal gluing is not a proper option for large corneal perforations (diameter ≥ 3 mm); instead, it necessitates therapeutic keratoplasty plus underlying disease management. The type of procedure used will depend on the extent and depth of the perforation: lamellar/full‐thickness graft or small‐diameter patch graft/large‐diameter keratoplasty. When gaps are not too extensive, tectonic grafts, also known as patch grafts, are used to fill corneal stromal defects, restore the structure of the cornea or sclera, and maintain the integrity of the globe. Lamellar or perforating patch grafts can be used permanently to repair peripheral perforations and descemetoceles, or they can be used as an acute measure to treat central corneal perforations (for potential future optical penetrating keratoplasty) [10]. Due to the large sterile perforation involving the center of the cornea in both eyes and the high risk of complications such as endophthalmitis, our patient underwent immediate sequential bilateral PKP. As far as we know, this is the first case of immediate sequential bilateral PKP. The 6‐month follow‐up results show anatomical and optical success. In Iran, corneal donor tissue is managed primarily through two Eye Bank centers, one affiliated with Mashhad University of Medical Sciences, where this case was treated. Our center prioritizes emergency cases such as corneal perforations, ensuring expedited tissue allocation. In this case, preserved donor corneas were used for both penetrating keratoplasties. However, the interval between graft harvesting and transplantation was less than 1 week, maintaining sufficient graft viability and transparency for therapeutic purposes. Given the satisfactory condition of the grafts and the absence of significant postoperative complications, no subsequent optical‐grade keratoplasty was required. Nonetheless, it is important to note that while emergency access to corneal tissue is generally reliable in our region, such access may vary in less‐equipped settings.

There are significant advantages and disadvantages of immediate sequential bilateral intraocular surgeries. The patient may face discomfort and visual loss challenges in the early postoperative period. Additionally, catastrophic complications such as endophthalmitis in cases of bilateral occurrence can result in permanent blindness and disability for the patient. In the presented case, the benefits of bilateral surgery outweigh its risks, given the bilateral corneal perforation and the high risk of complications.

## Author Contributions

**Javad Sadeghi:** conceptualization, investigation, project administration, writing – original draft, writing – review and editing. **Mohammadreza Sedaghat:** investigation, project administration, writing – review and editing. **Mitra Karimi Amir Abadi:** data curation, writing – original draft. **Mehrdad Motamed Shariati:** data curation, investigation, methodology, supervision, visualization, writing – original draft, writing – review and editing.

## Ethics Statement

We confirm that all interventions and screenings were carried out following relevant guidelines and regulations.

## Consent

The patient's legal guardian has given informed consent for the participation and publication of this case. The patient's identity, privacy, and confidentiality have been maintained. Written informed consent was obtained from the patient's legal guardian to publish this case report and any accompanying images.

## Conflicts of Interest

The authors declare no conflicts of interest.

## Data Availability

The datasets are available from the corresponding author upon formal and logical request.
